# Supplementary material for: Association between changes in social capital and mental well-being among older people in China
Source: PeerJ. 2022 Aug 25;10:e13938. doi: 10.7717/peerj.13938 (PMC9420402; doi:10.7717/peerj.13938)
Supplement: Supplemental Information 5 [file peerj-10-13938-s005.docx]

Table. scores of variable that measure social capital.

| **Variables** | **Score** | |
| --- | --- | --- |
| Whether you have pension and medical insurance | No | 0 |
|  | Either | 1 |
|  | Both | 2 |
| Whether you have a job | No | 0 |
|  | Yes | 1 |
| Who usually take care of you when you are unwell | Don't know | 0 |
|  | No one | 1 |
|  | Others | 2 |
|  | Other family members | 3 |
|  | Spouse or children | 4 |
| Where you usually go for medical treatment when you are ill | Don’t know | 0 |
|  | Not going to the doctor | 1 |
|  | Clinics | 2 |
|  | Township health center | 3 |
|  | Specialist hospitals | 4 |
|  | General hospital | 5 |
| Satisfaction with medical conditions | Don't know | 0 |
|  | Very dissatisfied | 1 |
|  | Dissatisfied | 2 |
|  | Generally | 3 |
|  | Satisfied | 4 |
|  | Very satisfied | 5 |
| Satisfaction of medical level | Don't know | 0 |
|  | Very dissatisfied | 1 |
|  | Dissatisfied | 2 |
|  | Generally | 3 |
|  | Satisfied | 4 |
|  | Very satisfied | 5 |
| Life satisfaction |  |  |
|  | Don't know | 0 |
|  | Very dissatisfied | 1 |
|  | Dissatisfied | 2 |
|  | Generally | 3 |
|  | Satisfied | 4 |
|  | Very satisfied | 5 |
| Evaluation of the local municipal government | Don't know | 0 |
|  | Worse | 1 |
|  | No grades | 2 |
|  | Some grades | 3 |
|  | A lot of grades | 4 |
| Confidence in their future | Don't know | 0 |
|  | Very unconfident | 1 |
|  | Unconfident | 2 |
|  | Generally | 3 |
|  | Confident | 4 |
|  | Very confident | 5 |
